# Influenza vaccine titer determination using biolayer interferometry (BLI)

**DOI:** 10.1186/1753-6561-9-S9-P75

**Published:** 2015-12-14

**Authors:** David W Wheatley, D Saunders, JH Welsh, E Matthews, IK Srivastava, MMJ Cox

**Affiliations:** 1 Pall Corporation, 5 Harbourgate Business Park, Southampton Road, Portsmouth, PO6 4BQ, UK; 2 Protein Sciences Corp, Meriden, USA

## Background

Fast, accurate determination of vaccine titer during influenza vaccine manufacture is important in understanding process performance and correctly scaling each process step. Traditionally Single Radial Immunodiffusion (SRID) assays have been used as the 'gold standard' but the assay requires very skilled operators to obtain reproducible results and is relatively low throughput. ELISAs have also been used to determine titer but have lower precision and dynamic range. BLI combines the high throughput characteristics of a 96-well plate based ELISA assay in conjunction with improvements in accuracy and repeatability derived from a simpler direct measurement of mass transfer on binding.

**Figure 1:**
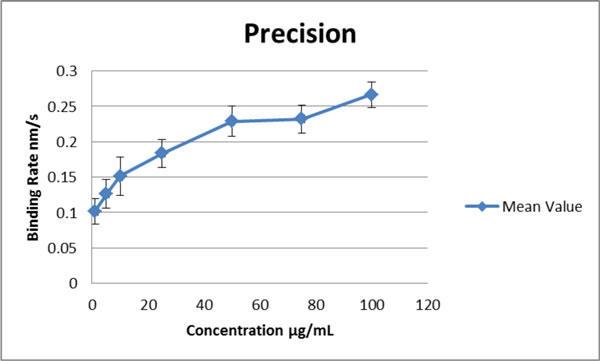
**CVs for each standard are <7 % in the range 10-100 μg/mL R2 values for each curve were >0**.97

The assay is based on the binding of the vaccine to polyclonal antibodies that recognise the influenza epitopes presented by the vaccine. The polyclonal antibody is bound to a protein G or protein A derivatized biosensor, depending on the animal source of the antibody. This configuration gives increased flexibility by allowing swift changes between vaccines derived from different viral strains by simply binding the paired antibody for the new strain to a biosensor without the need for derivatization. Hence the assay is suitable for the rapid changes in the viral strains represented in a vaccine. A robust assay, capable of determining vaccine titer from various process stages has been developed. Figure 1 illustrates coefficient of variance and binding rate across a range of concentrations. The assay has been shown to be applicable to both attenuated and synthetic vaccines and is an effective test for vaccine potency.

## Materials and methods

Samples taken directly from purification steps were assayed with zero sample clean up, having been only diluted with a proprietary ForteBio sample buffer. Protein A and Protein G biosensors were used in the assay. The system uses well plate technology allowing for high sample throughput.

## Results

Native samples gave an average result of 53.6 μg/mL. The heat treated samples showed a loss of response to an average value of 7.2 μg/mL, proving they had been inactivated and that the assay is also a test for vaccine potency.

## Conclusions

The total analysis time was less than 3 hours on the Octet RED96 System. The BLItz platform allowed at-line sampling to be conducted in the process development laboratories. A change in the vaccine strains or formulation did not require a change in equipment or method: only limited requalification of the assay would be required. In-process samples and purified samples were both analysed without matrix effect issues. The assay successfully analysed heat inactivated samples and is therefore a test for potency.

